# Positive Unanimous Voting Algorithm for Focal Cortical Dysplasia Detection on Magnetic Resonance Image

**DOI:** 10.3389/fncom.2016.00025

**Published:** 2016-03-29

**Authors:** Xiaoxia Qu, Jian Yang, Shaodong Ma, Tingzhu Bai, Wilfried Philips

**Affiliations:** ^1^Beijing Engineering Research Center of Mixed Reality and Advanced Display, School of Optics and Electronics, Beijing Institute of TechnologyBeijing, China; ^2^Department of Telecommunications and Information Processing (IPI-TELIN-iMinds), Ghent UniversityGhent, Belgium

**Keywords:** epilepsy, focal cortical dysplasia, magnetic resonance images, lesion detection

## Abstract

Focal cortical dysplasia (FCD) is the main cause of epilepsy and can be automatically detected via magnetic resonance (MR) images. However, visual detection of lesions is time consuming and highly dependent on the doctor's personal knowledge and experience. In this paper, we propose a new framework for positive unanimous voting (PUV) to detect FCD lesions. Maps of gray matter thickness, gradient, relative intensity, and gray/white matter width are computed in the proposed framework to enhance the differences between lesional and non-lesional regions. Feature maps are further compared with the feature distributions of healthy controls to obtain feature difference maps. PUV driven by feature and feature difference maps is then applied to classify image voxels into lesion and non-lesion. The connected region analysis then refines the classification results by removing the tiny fragment regions consisting of falsely classified positive voxels. The proposed method correctly identified 8/10 patients with FCD lesions and 30/31 healthy people. Experimental results on the small FCD samples demonstrated that the proposed method can effectively reduce the number of false positives and guarantee correct detection of lesion regions compared with four single classifiers and two recent methods.

## Introduction

**Figure 1:**
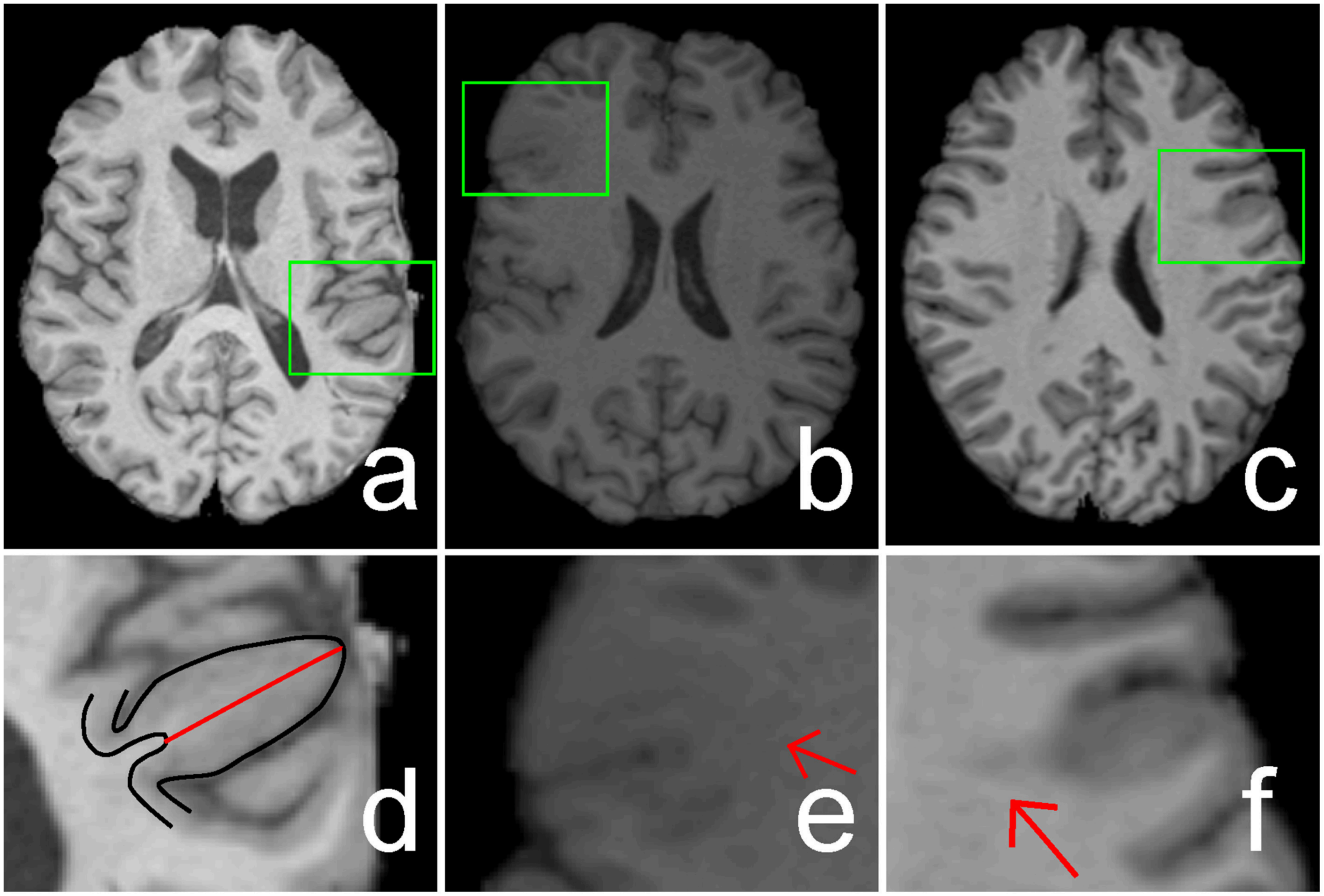
**Illustrations of cortical thickening (A), blurring of gray matter (GM)/white matter (WM) junction (B), and hyper-intensity signal (C) within focal cortical dysplasia (FCD) regions (indicated by green rectangles)**. The FCD regions in **(A–C)** are enlarged and shown in **(D–F)**, respectively. The black lines in image **(D)** are the boundaries of GM or cortex, whereas the length of the red line shows the increased cortical thickness. Image **(E)** shows that the GM and WM have a blurry boundary (indicated by a red arrow). Hyper-intensity signal within the WM region is indicated by a red arrow in image **(F)**.

Focal cortical dysplasia (FCD) is the main cause of epilepsy, which is a chronic illness of human brain that affects 50–65 million people worldwide (Bernasconi and Bernasconi, 2011). FCD, as a brain malformation of neocortical development, can be eliminated by respective surgery (Despotovic et al., 2011). Neurologists use magnetic resonance (MR) imaging as a non-invasive clinical tool during surgical planning to determine the location of the FCD lesion (Antel et al., 2003). The FCD volumes can be ranged from tiny size of 734 mm^3^ to large size of 80,726 mm^3^ (Colliot et al., 2006). The location of the FCD lesion is completely random in its cortical boundary distribution. Different patients have lesions at different locations within the cortex which has complex gyral structure. The lesion in the MR image exhibits three features, namely, cortical thickening, blurring of the gray/white matter (GM/WM) junction, and hyper-intensity signal within lesional region compared with other cortical regions (Figure 1; Bernasconi et al., 2001).

However, an MR image consists of many slices; for example, 175 sagittal slices exist for each MR image of one patient. Thus, visual detection of lesions is time consuming. Diagnosis results are prone to present large variances when the neurologist is tired because of heavy workload from observing MR images. The image-based diagnostic method is very much subjective, because it heavily depends on the neurologist's personal experience and knowledge.

Numerous studies have attempted to develop image processing methods to facilitate the detection and localization of FCD lesions to enhance the efficiency and accuracy for diagnosis of FCD lesions on MR images. Many feature maps have been proposed to enhance the contrasts of lesional and non-lesional regions. The following maps are used to detect distinct features: GM thickness map for cortical thickening (Antel et al., 2002); gradient map for blurring of GM/WM junction (Bernasconi et al., 2001) relative intensity map for hyper-intensity signal (Bernasconi et al., 2001), mean GM density map (Kassubek et al., 2002), junction image for blurring of GM/WM junction (Huppertz et al., 2005), and complex diffusion map for blurring of GM/WM junction and cortical thickening (Rajan et al., 2009). A new feature named GM/WM boundary (GWB) width map was developed to improve the detection of blurry GM/WM junction within FCD lesional region (Qu et al., 2014). All these feature maps have increased contrasts between lesional and non-lesional regions and can be utilized to assist lesion detection.

Several classification methods, such as threshold method (Kassubek et al., 2002; Huppertz et al., 2005), Bayesian classifier (Antel et al., 2003), support vector machine (Loyek et al., 2008), and neural network classifier (Besson et al., 2008a,b), have been applied to classify image voxels or regions into positives (lesional) and negatives (non-lesional) to automatically detect the lesion. Threshold methods consider the intensity of T1 MR data as a feature, compare the intensities of patients with those of healthy controls, and classify the voxels of images into lesional and non-lesional. The Bayesian classifier, support vector machine, and neural network classifiers do not only analyze the intensity feature but also consider advanced feature maps as classification criterion, such as GM thickness and gradient maps. These classification methods can correctly classify most of the FCD regions as positive, but numerous false positive (FP) regions are also produced (Besson et al., 2008a,b). Moreover, the shapes, locations, and sizes of the FCD lesions remarkably vary in patients. Thus, automated FCD detection remains a challenge.

In this study, we developed a new framework for positive unanimous voting (PUV) to reduce the FP regions for automatic FCD detection. Unanimous voting combines multiple classifiers, which can be used to improve the performance (such as classification errors) of single classifiers (Wozniak et al., 2014). The proposed framework mainly consists of five parts, namely, image normalization, feature determination, unanimous voting for feature classification, region connection analysis, and evaluation.

Image normalization improves the image quality for subsequent study. In feature determination, maps for GM thickness, gradient, relative intensity, and GWB were considered features for distinguishing lesional and non-lesional voxels. In addition, feature distributions of healthy controls were integrated into the computation of feature differences between test images and healthy controls.

Unanimous voting involves the classification of image voxels into lesional or non-lesional. Multiple classifiers were previously combined to improve the performance of single classifiers (Wozniak et al., 2014). These combinations mainly include majority voting, voting of one against all, and unanimous voting. We selected unanimous voting with respect to positive results to combine multiple classifiers to reduce the FP. Feature value distributions of lesional and non-lesional regions were overlapped. Thus, in this study, the decision function of classification was constructed utilizing the naïve Bayesian (NB), linear discriminant analysis (LDA), Mahalanobis discriminant analysis (MDA), and quadratic discriminant analysis (QDA) classifiers (Duda et al., 2000), which were chosen as the basic classifiers for optimization.

Considering that the high similarity in features of lesional and non-lesional regions, the basic classifiers are comprised of the classifiers that calculate both the mean and the variance in each class. In addition, these classifiers are as stable in convergence as the support vector machine and neural network based methods are. A large false positive rate can cause classifiers based on support vector machine or neural network methods not be able to converge. Moreover, for this particular application the parametric estimation of the support vector machine and neural network methods can be overly complicated. Therefore, the NB, LDA, MDA, and QDA classifiers were chosen as basic classifiers for detection.

Connected region analysis was designed to remove tiny fragment regions and allow the classification to extend into the subject level. Each classified image contained voxels falsely classified as positive. These voxels formed irregular small areas called fragment regions. Connected region analysis measured the size values of these tiny fragment regions. Regions smaller than a pre-set value were relabeled as negative. In subject level classification, each subject was recognized as patient when the refined image of the subject contained voxels classified as positive (lesional). Otherwise, the subject was considered healthy.

The true positive (TP) rate (TPR), FP rate (FPR), precision, recall, and F score (*F*_s_) were chosen as evaluation metrics to evaluate the performance of the proposed methods. The evaluation values were computed by comparing the classified images with the ground truth. Classification results of the different feature groups were compared to validate the effectiveness of the features. To demonstrate the performance of the proposed method, it was compared with single classifiers, such as NB, LDA, MDA, and QDA, in terms of the classified results. Moreover, the two-stage Bayesian classifier (TSBC; Antel et al., 2003) and surface-based LDA (SLDA; Hong et al., 2014) methods were also evaluated using the same metrics as the proposed method.

The main contributions of this work could be summarized as follows. (1) A detection framework using positive results based on unanimous voting of multiple classifiers was proposed to classify images into lesional and non-lesional. Thus, the detection results provided less FP voxels than single classifier-based methods. Experimental results clearly demonstrated that the proposed work was more effective than the single classifiers and the two state-of-the-art methods for FCD detection. (2) Mean representations of the healthy model were integrated into feature determination, which could differentiate lesional and non-lesional regions more efficiently. For example, the averaged F score resulted from different classifiers using FG6 is bigger than which using FG3 (0.071 vs. 0.052). Here, the FG6 integrates the mean representations of the healthy model, while FG3 does not. (3) Connected region analysis removed tiny FP fragments and extended the evaluation of the voxel level to the subject level.

## Methods

**Figure 2:**
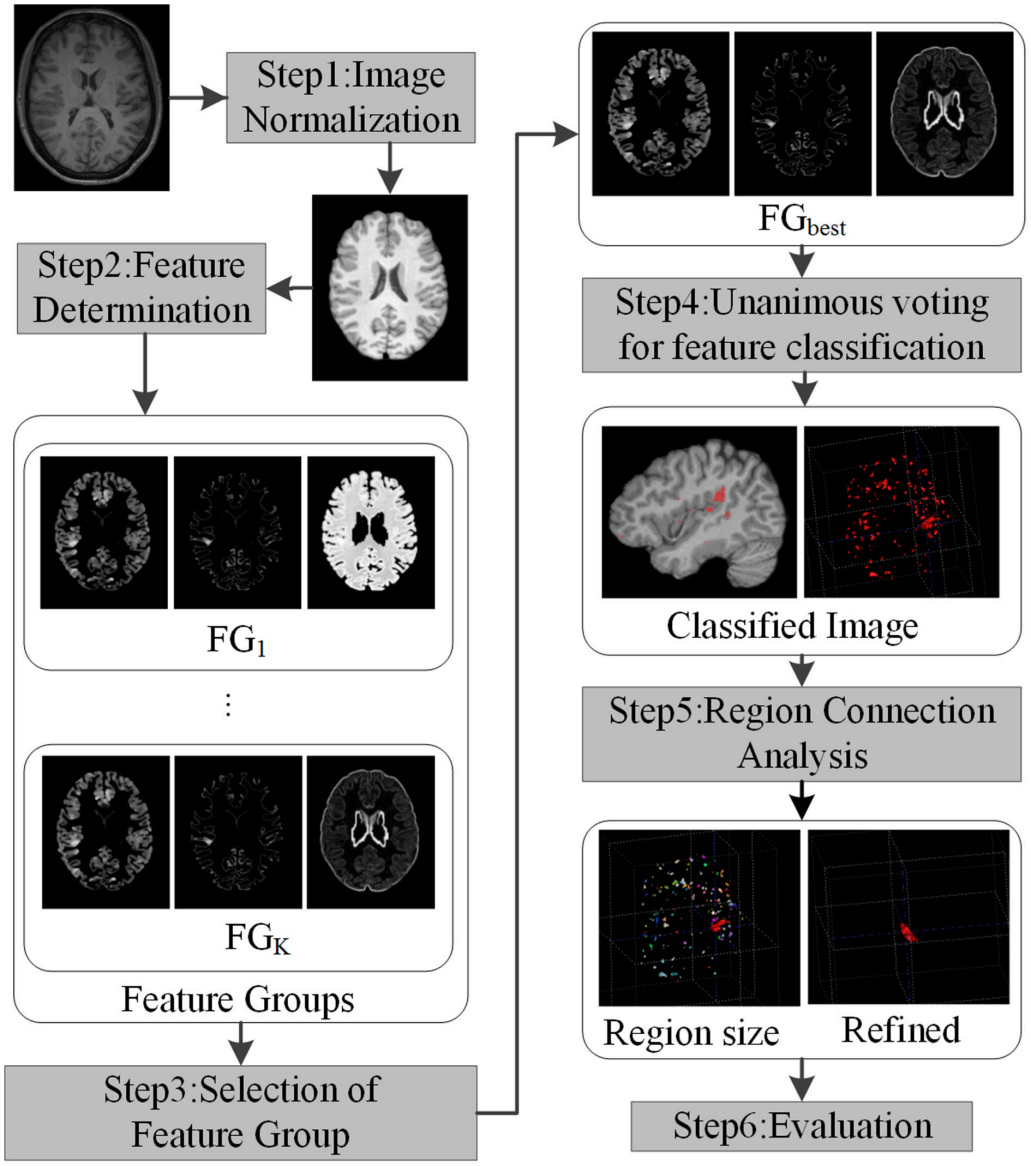
**Framework of the proposed work**. Each step is indicated by rectangles with grayscale shade.

The framework for detecting FCD lesion on T1-weighted MR image is illustrated in Figure 2. The framework involved five major processing steps. (1) MR images were normalized. (2) Features were determined to compute and group features, which enhanced the differences between lesional and non-lesional regions. The feature groups were then evaluated and selected to establish the best group for further analysis. (3) Unanimous voting driven by the selected best feature group (FG_best_) for feature classification was performed to classify the images into lesional and non-lesional regions. (4) Connected region analysis was utilized to reclassify the falsely classified tiny fragment regions into negative to refine the classified results. (5) Refined images were evaluated as the final results.

### Image normalization

Image standardization (Nyul and Udupa, 1999) was performed to refine the original raw image to a predefined scale ranging from 0 to 255. The original images were first oriented into the right-posterior-inferior direction. The brain extraction tool proposed by Smith et al. (Smith, 2002) was applied to extract the brain region on the oriented images because FCD lesions only occur in brain regions. Intensity non-uniformity (also called bias field) was corrected using the expectation–maximization algorithm (Zhang et al., 2001) on all images to obtain consistent intensities of the extracted brain region.

Each histogram of the 3D images has two peaks which correspond to gray matter and white matter regions, respectively. To insure all the images are normalized properly, we took one standardized image of healthy people as reference. When the areas under the two peaks on the histograms of the query and the reference images were almost overlapped (90% overlapped), the intensities of the query image were taken as normalized adequately.

After the intensity normalization, the space related normalization was performance. We tri-linearly interpolated images into the same resolution to standardize voxel size. We used rigid registration to roughly align the images and unify the space of the images, followed by affine registration to further standardize the images. The reference image for registration was the MNI152 brain T1-weighted MR image from the Montreal Neurological Institute (Montreal Neurological Institute). We could only align general brain regions, not match details such as gyral GM regions, because brain structures of different subjects exhibit distinct topological structures. Thus, we could scale the brain regions into similar sizes while maintaining the topological structure and details. Consequently, differences in total brain volume between various subjects were adjusted. Brain tissues that were not anatomically related to FCD lesions (e.g., brain cerebellum, brain stem, striatum, and thalamus) were removed from the registered images. The brain atlas of images from MNI (Mazziotta et al., 2001; Diedrichsen et al., 2009) was considered the template for elimination.

### Feature determination

Feature determination was used to compute feature maps, which could enhance contrasts between lesional and non-lesional regions. Feature determination involved four steps. First, features were computed from MR images. Second, average feature representations of healthy controls were calculated. Third, feature differences between the test images and healthy controls were generated. Fourth, feature groups were selected.

The brain tissues were first segmented to acquire tissue space information before the feature maps were computed. We segmented the brain data into partial volume maps of GM, WM, and cerebrospinal fluid using a hidden Markov random field model and expectation-maximization algorithm to consider the effect of partial volume (Zhang et al., 2001).

**Figure 3:**
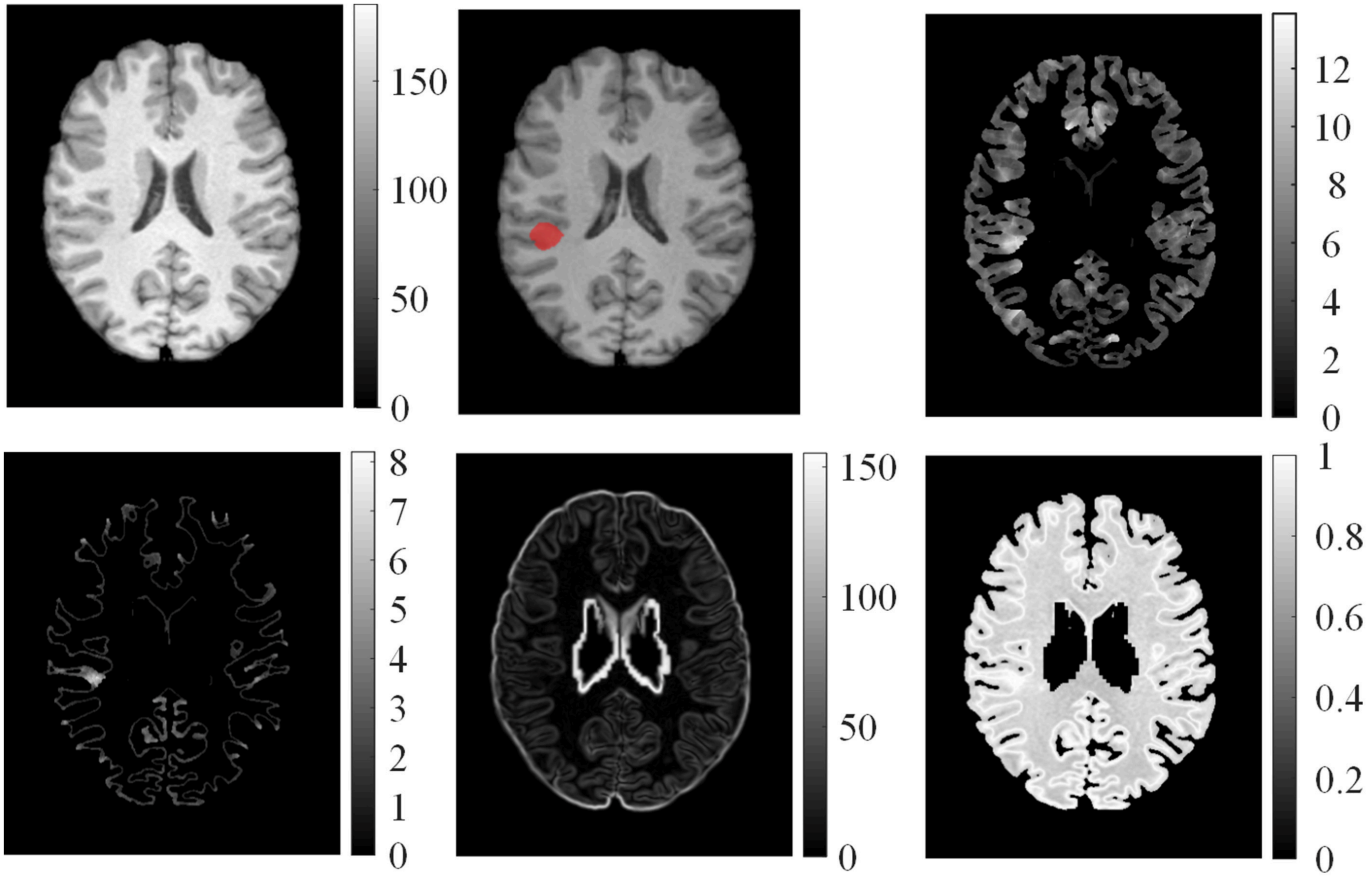
**Examples of features computed from T1 MR images**. From left to right on the top row: normalized image, ground truth where the FCD lesion is labeled in red, and GM thickness map. On the bottom row: GM/WM boundary (GWB) width map, gradient map, and relative intensity map.

We computed the GM thickness map (denoted by *F*_1_), gradient map (*F*_2_), relative intensity map (*F*_3_; Bernasconi et al., 2001; Antel et al., 2002), and GWB width map (*F*_4_; Qu et al., 2014) for each individual subject on the pre-processed images to capture the features of FCD regions in the MR images. These features formed a feature vector ***F*** = {*F*_1_, *F*_2_, *F*_3_, *F*_4_}, and they are shown in Figure 3. Lesional regions had larger values than non-lesional regions on the GM thickness map, GWB width map, and relative intensity map. The gradient map and lesional regions with blurring of GWB had lower values than the non-lesional regions because lower values indicate blurry regions.

**Figure 4:**
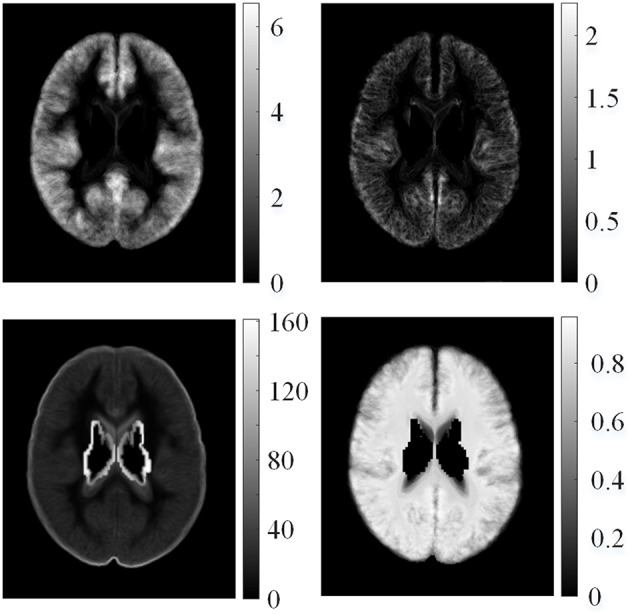
**Examples of mean features of healthy controls**. From left top row, clockwise: mean GM thickness map, mean GWB width map, mean gradient map, and mean relative intensity map of healthy controls.

Average feature representations of healthy controls referred to the mean values in sliding local windows of images. Images of different subjects could be compared because the images of the subjects were registered into the same MNI space using image normalization (Section Image Normalization). The mean model of healthy controls for the *i*-th feature map *F*_*i*_ is as follows:

(1) $$\begin{array}{r} F_{\mu ,i}\left(v\right)=\frac{1}{K\cdot N}\overset{k}{\underset{k=1}{\sum }}\left(\overset{N}{\underset{n=1}{\sum }}F_{i}^{\left(k\right)}\left(v_{n}\right)\right) \end{array}$$

where *F*_μ, *i*_(***v***) is the average value for the *i*-th feature map of all healthy controls at voxel ***v***, ***v***_*n*_ is the coordinate of the *n*-th neighboring voxel within a 3D local window centered on voxel **v**, *N* is the total number of neighboring voxels, $F_{i}^{\left(k\right)}\left(v_{n}\right)$ is the feature value of the *k*-th healthy control at voxel ***v***_*n*_, and *K* is the total number of healthy controls. The example of mean features of healthy controls is shown in Figure 4. The feature value distributions of healthy controls were not uniform, that is, they varied in different locations in the human brain.

**Figure 5:**
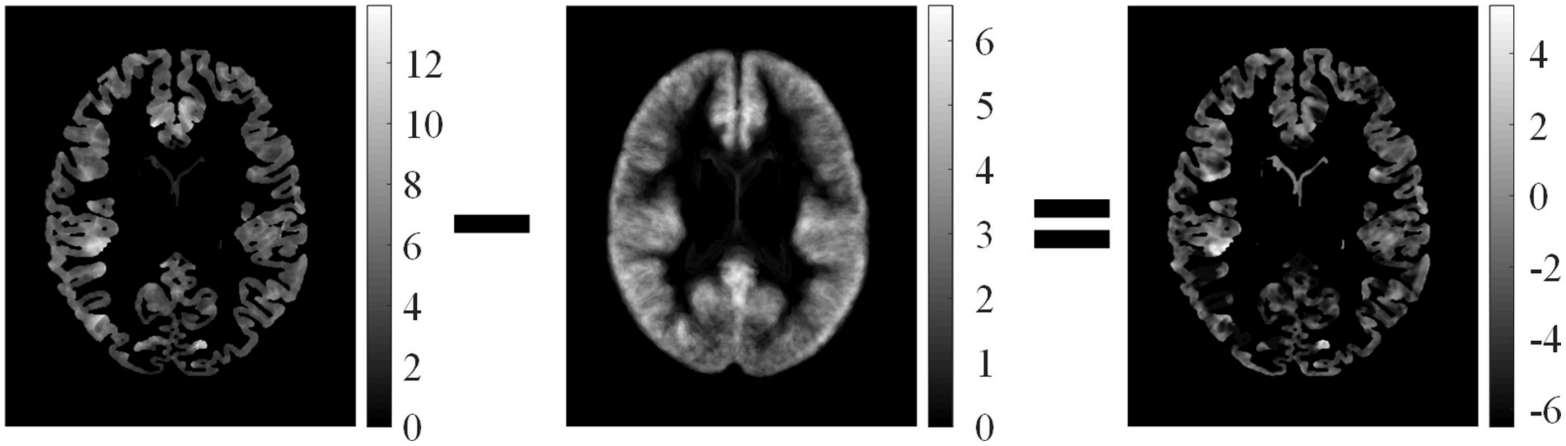
**Illustrations of computed feature differences between test images and healthy controls**. From left to right: GM thickness map of one patient on axial view, mean values of GM thickness map of healthy controls, and feature difference in GM thickness map.

Feature difference measured variations between feature ***F*** and average feature of healthy controls *F*_μ, *i*_. The procedure is illustrated in Figure 5. *F*_*d*1_ eliminated the influence of distributions on healthy controls. Thus, the feature difference of GM thickness map denoted by *F*_*d*1_ showed the increased cortical thickness better than the GM thickness *F*_1_. The feature difference was denoted by vector ***F***_*d*_ = {*F*_*d*1_, *F*_*d*2_, *F*_*d*3_, *F*_*d*4_}, and it can be computed as follows:

(2) $$\begin{array}{r} F_{di}\left(v\right)={\text{F}}_{i}\left(v\right)-{\text{F}}_{\mu ,i}\left(v\right) \end{array}$$

**Figure 6:**
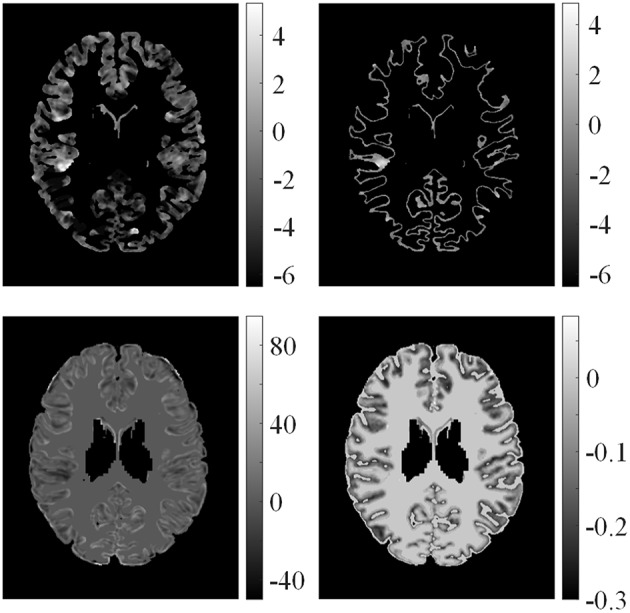
**Examples of feature differences between test images and healthy controls**. From left top row, clockwise: feature differences of GM thickness map, GWB width map, gradient map, and relative intensity map from one patient.

Examples of feature differences between the test images and healthy controls were generated and are illustrated in Figure 6. The feature difference ***F***_*d*_ images enhanced contrasts between lesional and non-lesional regions compared with feature ***F***.

Features and feature differences were then grouped according to their actual meanings, such as detecting cortical thickening or measuring the relative cortical thickening in contrast to healthy controls, to determine a good combination of features for classification (Table 1). Feature group 1 (FG_1_) included three basic FCD features, namely, maps of GM thickness, gradient, and relative intensity, which had been proven to be effective for FCD detection (Antel et al., 2003). We assembled the features into FG_2_ and FG_3_ to evaluate the performance of the GM/WM width map with respect to FCD detection. FG_1−3_ were features in which the feature distributions of healthy models were not considered, whereas FG_4−6_ were feature differences that included the influence of healthy controls.

**Table 1:** **Feature Groups**.

	***F*_1_**	***F*_2_**	***F*_3_**	***F*_4_**		***F*_***d***1_**	***F*_***d***2_**	***F*_***d***3_**	***F*_***d***4_**
FG_1_	+	+	+	–	FG_4_	+	+	+	–
FG_2_	+	+	+	+	FG_5_	+	+	+	+
FG_3_	+	–	+	+	FG_6_	+	–	+	+

“*+” means that the feature is present in the feature group, “−” indicates absence of feature. F_1_, F_2_, F_3_, and F_4_ are the maps of gray matter (GM) thickness, gradient, relative intensity, and gray/white matter boundary width map. F_d1_, F_d2_, F_d3_, and F_d4_ are the corresponding feature differences of F_1_, F_2_, F_3_ and F_4_*.

**Figure 7:**
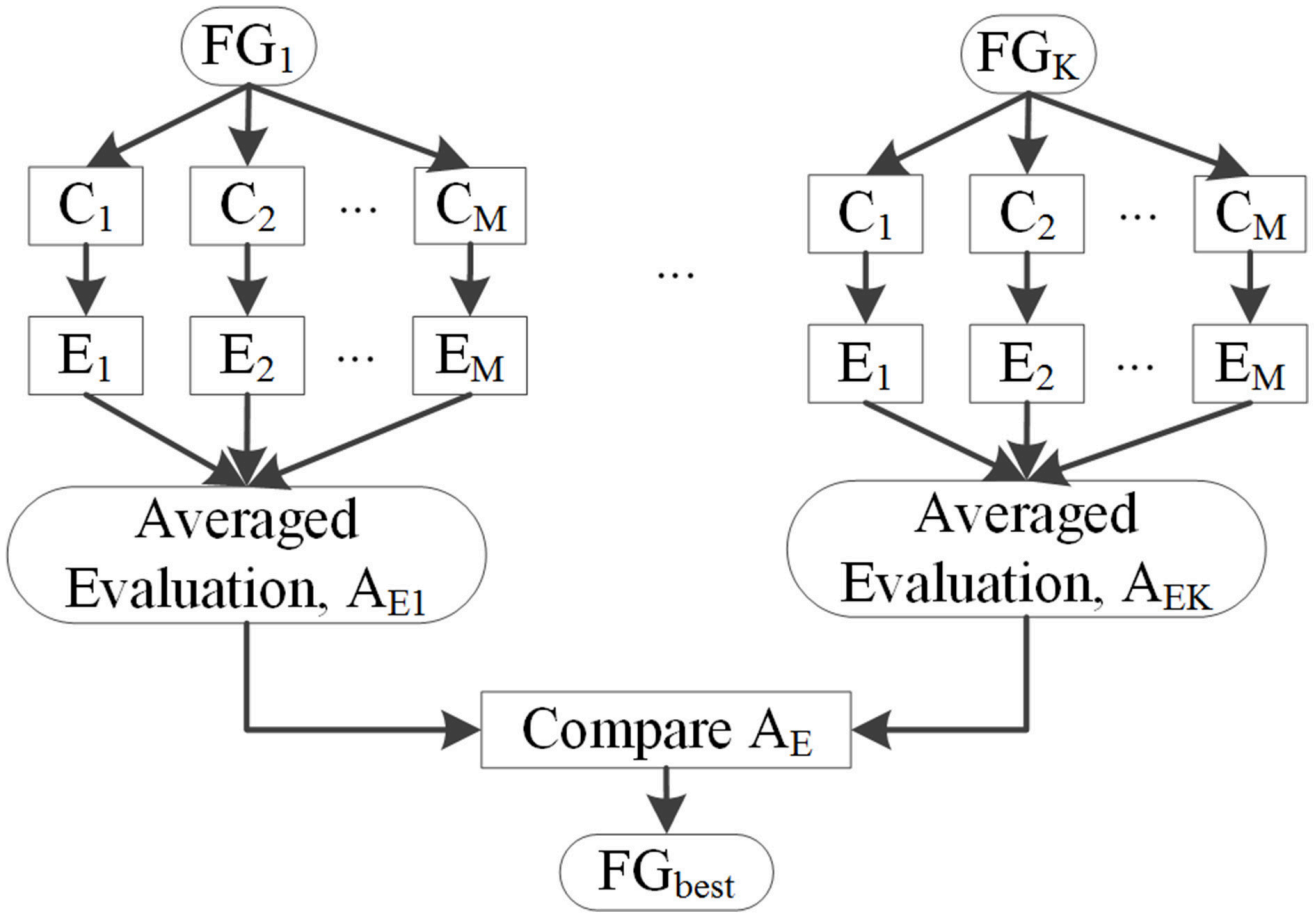
**Selection of feature groups**.

Several classifiers, denoted by *C*_1_, *C*_2_, …, *C*_*M*_, were used to classify image voxels driven by different feature groups FG_k_ to select FG_best_. The results of each classifier were evaluated by *F*_s_, and the resulting *E*_m_ (m = 1, 2 …, M) is shown in Figure 7. Evaluation values of different classifiers for each feature group were averaged, with the resulting average value denoted by *A*_*EK*_(*k* = 1, 2, …, *K*), where *k* is the index of the feature group. FG_best_ was the feature group with the highest value of A_Ek_. Classifiers *C*_1_, *C*_2_, …, *C*_*M*_ were the NB, LDA, MDA, and QDA classifiers, which were also applied for classification using unanimous voting for feature classification and will be introduced in Section Unanimous Voting for Feature Classification.

### Unanimous voting for feature classification

**Figure 8:**
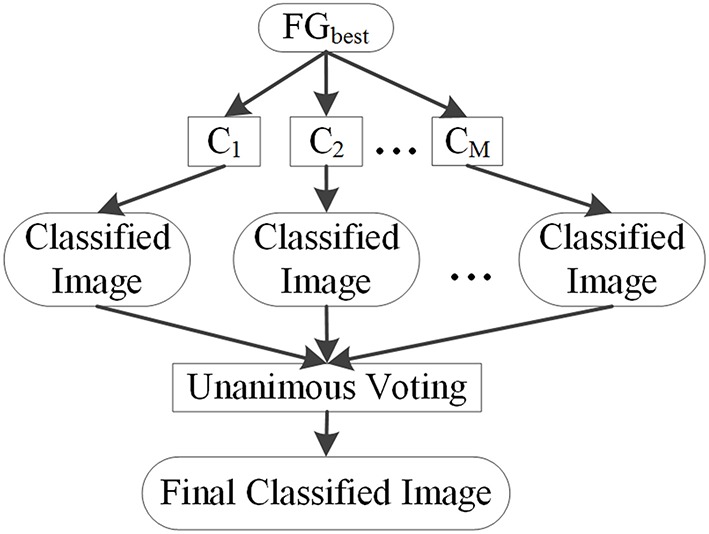
**Basic principle of unanimous voting for feature classification**.

Unanimous voting for feature classification was performed to classify image voxels into lesional and non-lesional using the proposed PUV method. The basic principle of this step is explained in Figure 8. The features in FG_best_ were the inputs for this step. Image voxels were first classified by basic classifiers *C*_1_, *C*_2_,…,*C*_*M*_. Then, unanimous voting of the classified images was performed to reclassify voxels falsely identified as positive into negative. We attempted to obtain an object function to achieve unanimous voting for feature classification instead of classifying images on multiple stages to simplify the procedure.

Each voxel ***v*** of an image was classified into two classes, namely, positive (lesional) and negative (non-lesional), denoted by *w*_1_ and *w*_2_, respectively. The feature vector of each voxel is denoted by ***x*** = {*x*_1_, *x*_2_, …, *x*_*f*_}, where ***x*** is composed of feature ***F*** and feature difference ***F***_*d*_. Each voxel was classified based on its feature vector.

The NB classifier model is expressed as follows:

(3) $$\begin{array}{l} p_{NB}\left(w_{i}|x\right)=p_{NB}\left(x|w_{i}\right)p_{NB}\left(w_{i}\right)∕\left(\sum p_{NB}\left(x|w_{i}\right)p_{NB}\left(w_{i}\right)\right), \end{array}$$

where *p*_*NB*_ (***x***|*w*_*i*_) is the likelihood of *w*_*i*_ with respect to ***x***, *p*_*NB*_ (*w*_*i*_) is the prior probability, and *p*_*NB*_ (*w*_*i*_|***x***) is the posterior probability. Likelihood was computed as follows:

(4) $$\begin{array}{l} p_{NB}\left(x\text{|}w_{i}\right)=\prod_{f=1}^{d}{\left(2\pi \sigma_{f,w_{i}}\right)}^{-0.5}\exp(-\left(x_{f}-{\text{μ}}_{f,w_{i}}\right)/\\ \;\left(2{\left(\sigma_{f,w_{i}}\right)}^{2}\right)), \end{array}$$

where μ_*f*,_*w*__*i*__ and σ_*f*,_*w*__*i*__ are the mean and standard deviation values of the *f* -th feature with respect to class *w*_*i*_, respectively. Posterior probability of the *LDA* is as follows:

(5) $$\begin{array}{l} p_{LDA}\left(w_{i}|x\right)=\mu_{w_{i}}{\text{Σ}}^{-1}x^{T}-\frac{1}{2}\;\mu_{w_{i}}\;{\text{Σ}}^{-1}\;\mu_{w_{i}}^{T}+ln\left(p_{LDA}\left(w_{i}\right)\right), \end{array}$$

whereμ_*w*_*i*__ is the mean of class *w*_*i*_, and Σ is the pooled covariance matrix. The posterior probability of the *QDA* classification is as follows:

(6) $$\begin{array}{l} p_{QDA}\left(w_{i}|x\right)=\mu_{w_{i}}\;{\text{Σ}}_{w_{i}}^{-1}\;x^{T}\;-\;\frac{1}{2}\;\mu_{w_{i}}\;{\text{Σ}}_{w_{i}}^{-1}\;\mu_{w_{i}}^{T}+ln\left(p_{QDA}\left(w_{i}\right)\right), \end{array}$$

where Σ_*w*_*i*__ is the variance matrix of class *w*_*i*_. The Mahalanobis distance between sample *x* and class *w*_*i*_ in the *MDA* classifier is as follows:

(7) $$\begin{array}{l} D_{MDA}\left(w_{i},x\right)=\sqrt{{\left(x-\mu_{w_{i}}\right)}^{T}{\text{Σ}}^{-1}\left(x-\mu_{w_{i}}\right)}. \end{array}$$

We reclassified the voxels identified as positive by the basic classifiers as negative or positive, because the results of basic classifiers contain a large number of FPs. The class labels *L*_*j*_(*v*)were defined as 1, if *v* ∈ *w*_1_. Otherwise, *L*_*j*_(*v*) = 0. *v* is the location of the 3D voxel in the MR image, and *j* is the index of the classifier. *L*_1_, *L*_2_, *L*_3_, and *L*_4_ are labels of classifiers NB, LDA, QDA, and MDA, respectively. The combination of multiple classifiers using unanimous voting can be formulated as follows:

(8) $$\begin{array}{l} L_{PUV}\left(\text{v}\right)=\overset{N}{\underset{j=1}{\prod }}L_{j}\left(\text{v}\right) \end{array}$$

where *N* is the total number of classifiers. When *L*_1_, *L*_2_,*L*_3_ and *L*_4_ were all labeled as 1, *L*_*PUV*_(*v*) was set to 1. Otherwise, *L*_*PUV*_(*v*) was set to 0. The final labeled *L*_*PUV*_(*v*) is defined as follows:

(9) $$\begin{array}{l} \begin{array}{c} {\;L}_{PUV}\left(\text{v}\right)=sign\left(p_{NB}\left(w_{1}|x\right)∕p_{NB}\left(w_{2}|x\right)\right)\\ ∙sign\left(p_{LDA}\left(w_{1}|x\right)-p_{LDA}\left(w_{2}|x\right)\right)\\ ∙sign\left(p_{QDA}\left(w_{1}|x\right)-p_{QDA}\left(w_{2}|x\right)\right)\\ ∙sign\left(p_{MDA}\left(w_{1}|x\right)-p_{MDA}\left(w_{2}|x\right)\right) \end{array} \end{array}$$

which can be calculated as follows:

(10) $$\begin{array}{l} L_{PUV}\left(v\right)=sign\left(\prod_{f=1}^{d}\frac{\sigma_{f,w_{2}}}{\sqrt{\sigma_{f,w_{1}}}}\text{exp}\left(\frac{x_{f}-\mu_{f,w_{2}}}{2\sigma_{f,w_{2}}^{2}}-\frac{x_{f}-\mu_{f,w_{1}}}{2\sigma_{f,w_{1}}^{2}}\right)\right)\\ •sign\left(\left({\text{μ}}_{{\text{w}}_{1}}\text{​}-\text{​}{\text{μ}}_{{\text{w}}_{2}}\right)\sum^{-1}x^{\text{T}}-\frac{1}{2}\left({\text{μ}}_{{\text{w}}_{1}}\Sigma^{-1}{\text{μ}}_{{\text{w}}_{1}}^{\text{T}}+{\text{μ}}_{{\text{w}}_{2}}\Sigma^{-1}{\text{μ}}_{{\text{w}}_{2}}^{\text{T}}\;\right)\right)\\ •sign\left(\mu_{w_{1}}\Sigma_{w_{1}}^{-1}\left(x^{T}-\frac{1}{2}\mu_{w_{1}}^{T}\right)-\mu_{w_{2}}\Sigma_{w_{2}}^{-1}\left(x^{T}-\frac{1}{2}\mu_{w_{2}}^{T}\right)\right)\\ •sign\left(\begin{array}{l} \sqrt{{\left(x-\mu_{w_{2}}\right)}^{T}\Sigma^{-1}\left(x-\mu_{w_{2}}\right)}/\\ \;\sqrt{{\left(x-\mu_{w_{1}}\right)}^{T}\Sigma^{-1}\left(x-\mu_{w_{1}}\right)} \end{array}\right) \end{array}$$

Equation (10) is the final classification decision of the proposed *PUV* method. Similar to Equation (8), when *L*_*PUV*_(*v*) was equal to 1, *v* was labeled as *w*_1_. Otherwise, *L*_*PUV*_(*v*) was equal to 0, and *v* was labeled as *w*_2_.

### Region connection

Region connection analysis was performed to refine the voxel-based classification results by removing the tiny fragmented regions. This process extended the classification to the subject level, in which each subject was recognized as either a patient or healthy person. This analysis was performed as follows. First, lesional regions in the classified images were labeled as 1, whereas healthy regions were labeled as 0. Second, the morphological opening with local window of *L*_*w*_ × *L*_*w*_ × *L*_*w*_ was then used to process regions labeled as 1, such that small noise voxels were removed, and weak connected regions were separated. Third, we labeled every connected region uniquely and measured the size of each connected region by counting the number of voxels. Fourth, lesional regions smaller than a threshold *T*_*s*_ were relabeled as non-lesional. Otherwise, the regional label was retained. Thus, tiny fragmented regions were removed from the lesional results.

Each subject was classified as either a healthy control or a patient after the tiny fragments were removed. If a subject's image contained voxels classified as lesional, the subject was considered a patient. Otherwise, the subject was considered a healthy control. Thus, the classification was extended from the voxel level to the subject level.

### Evaluation

We compared the classified images against the ground truth using the evaluation metrics, namely, TPR, FPR, and *F*_s_, to evaluate the performance of the classification methods. Evaluations were composed of voxel- and subject-based evaluation. Voxel evaluation computes the number of correctly and incorrectly classified voxels, whereas subject evaluation calculates the number of correctly and incorrectly classified subjects. We analyzed voxel-based evaluation as an example, and the results are discussed below.

First, a TP is a correctly identified lesional voxel, whereas an FP is a non-lesional voxel incorrectly identified as a lesional voxel. A false negative (FN) is a lesional voxel incorrectly identified as a non-lesional voxel, whereas a true negative (TN) is correctly identified as a non-lesional voxel.

Second, the TPR defines how many correct positive results occur among all positive samples, that is, TPR = #TP∕(#TP + #TN) (hereafter, # means the number of the parameter cited). FPR defines the number of incorrect positive results that occur among all negative samples, that is, FPR = #FP∕(#FP + #TN).

Third, we used *F*_s_ to evaluate the trade-off between precision and recall. Precision is the probability that the positive results are TP and defined as precision = #TP∕(#TP + #FP). Recall (similar to TPR) indicates the percentage of positive regions in ground truth classified as positive. *F*_s_ can be interpreted as the weighted average of precision and recall and defined as *F*_s_ = 2 × precision × recall∕(precision + recall). *F*_s_ reaches its best value at 1 and worst score at 0. A larger *F*_s_ indicates better trade-off between precision and recall.

## Experimental results and discussion

### Experimental data

We studied the T1-weighted MR images of 10 patients with FCD lesions and 31 healthy controls (one image per subject). Images were acquired at Ghent University Hospital on a Siemens 3T MR scanner. Each image consisted of 256 × 256 × 176 voxel matrices with a resolution of 0.8594 × 0.8594 × 0.9 mm. A doctor manually delineated the FCD lesions in the images of FCD patients prior to this study. All subjects were processed to be anonymous before this study to protect their privacy. The data used in this study were extracted from a retrospective study that was approved by the local Ethics Committee of the Ghent University Hospital. The 10 patients and 31 healthy people involved in our study have provided written consent. All patients suffered from epilepsy due to FCD have been confirmed by clinical examinations.

### Experimental design

**Figure 9:**
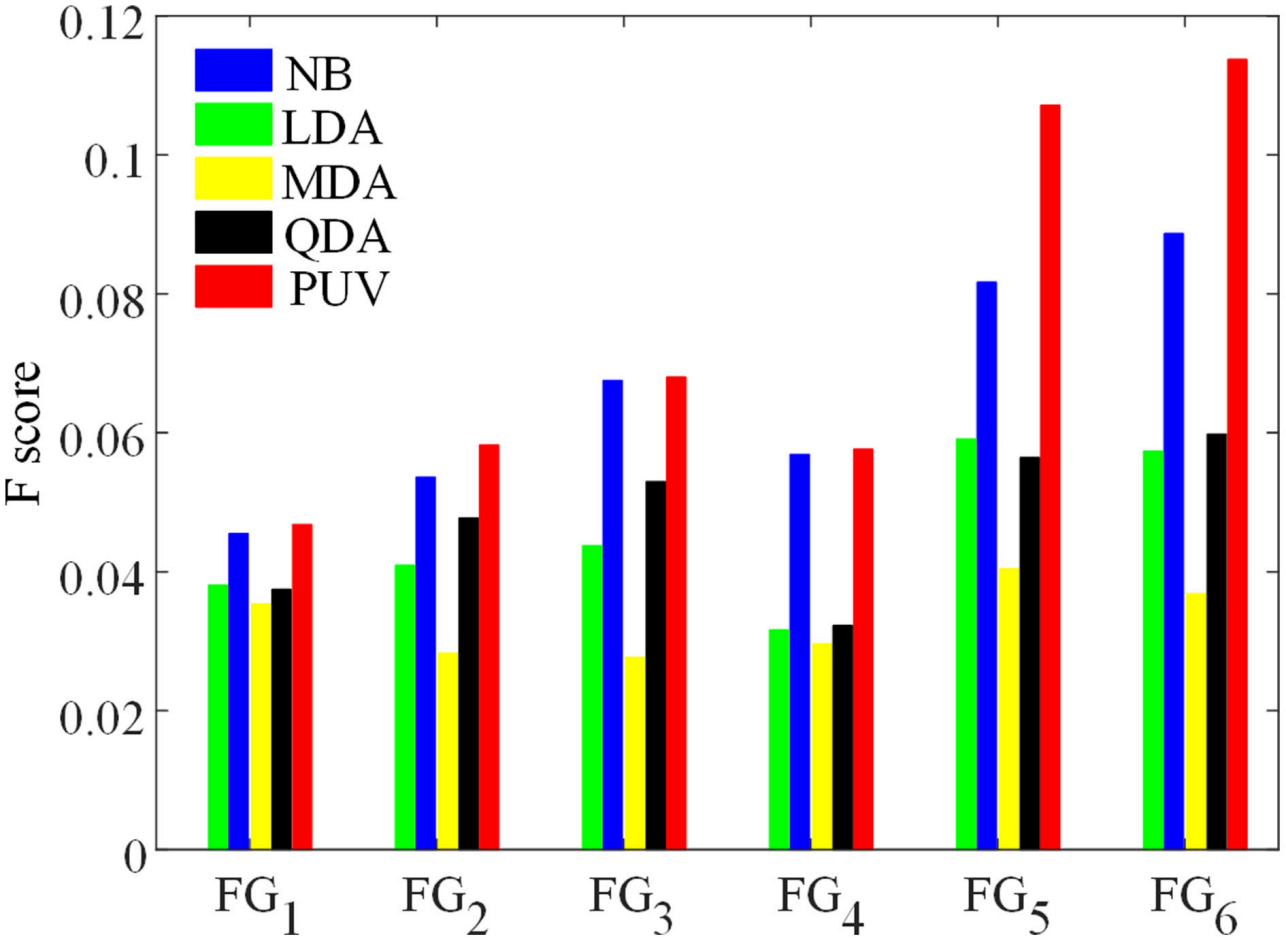
**Comparison of F-scores of the classification results from different feature groups and classification methods**. The feature groups FG_1−6_ are defined in Table 1. FG_1_ includes maps of GM thickness, gradient, and relative intensity. FG_2_ includes maps in FG_1_ and the GM/WM width map. FG_3_ includes maps of GM thickness, relative intensity and GM/WM width. The values of FG_4−6_ are feature differences that included the influence of healthy controls, and corresponded to the FG_1−3_, respectively.

**Figure 10:**
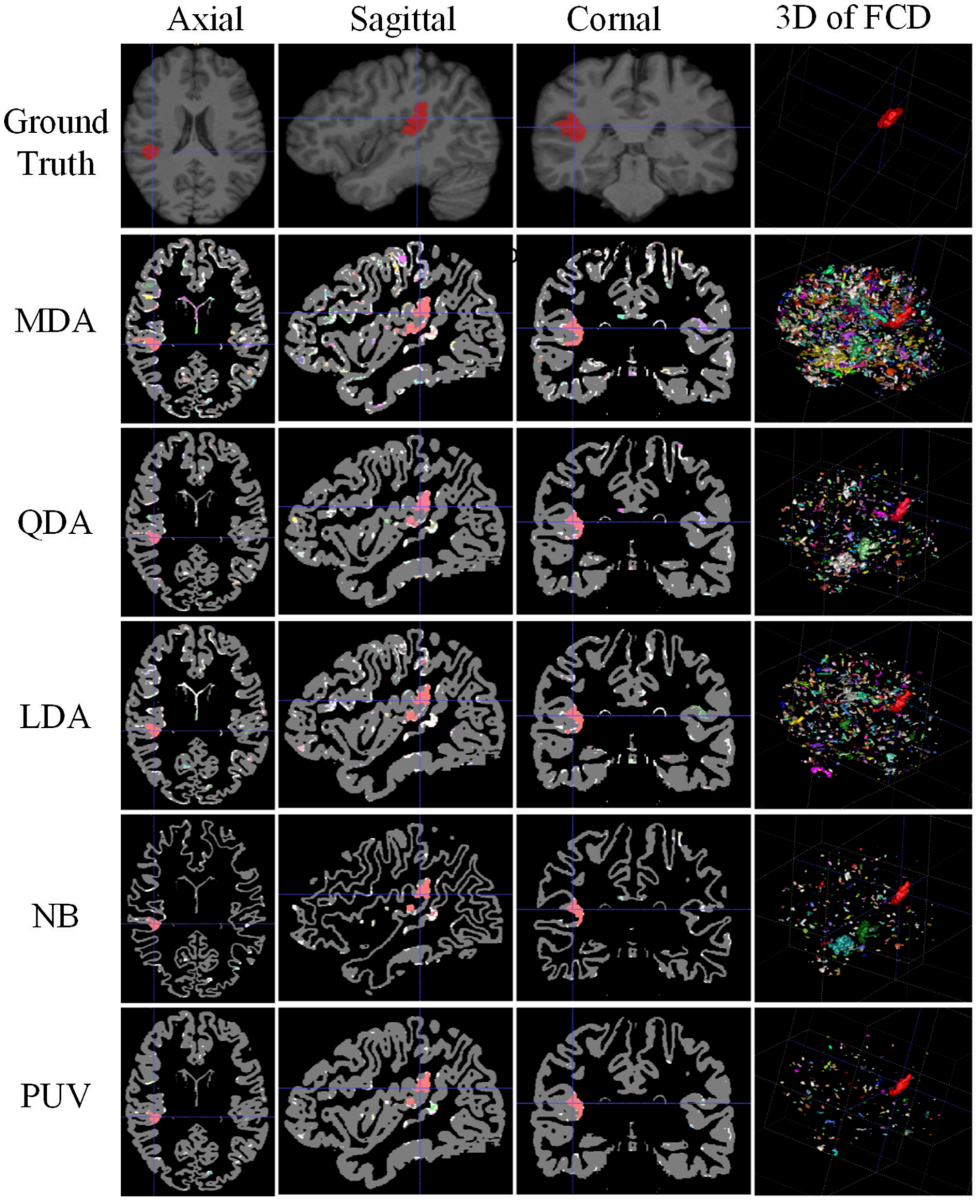
**Comparison of examples of classified images from positive unanimous voting (PUV) and single classifiers used in this study**. From top to bottom, images of each row are ground truth and classified images from Mahalanobis discriminant analysis, quadratic discriminant analysis, linear discriminant analysis, naïve Bayesian, and PUV. From left to right, columns show axial, sagittal, coronal, and 3D views. For illustration purposes, the different connected regions of positive results are described by different colors on the 3D views. FCD regions are colored red.

**Figure 11:**
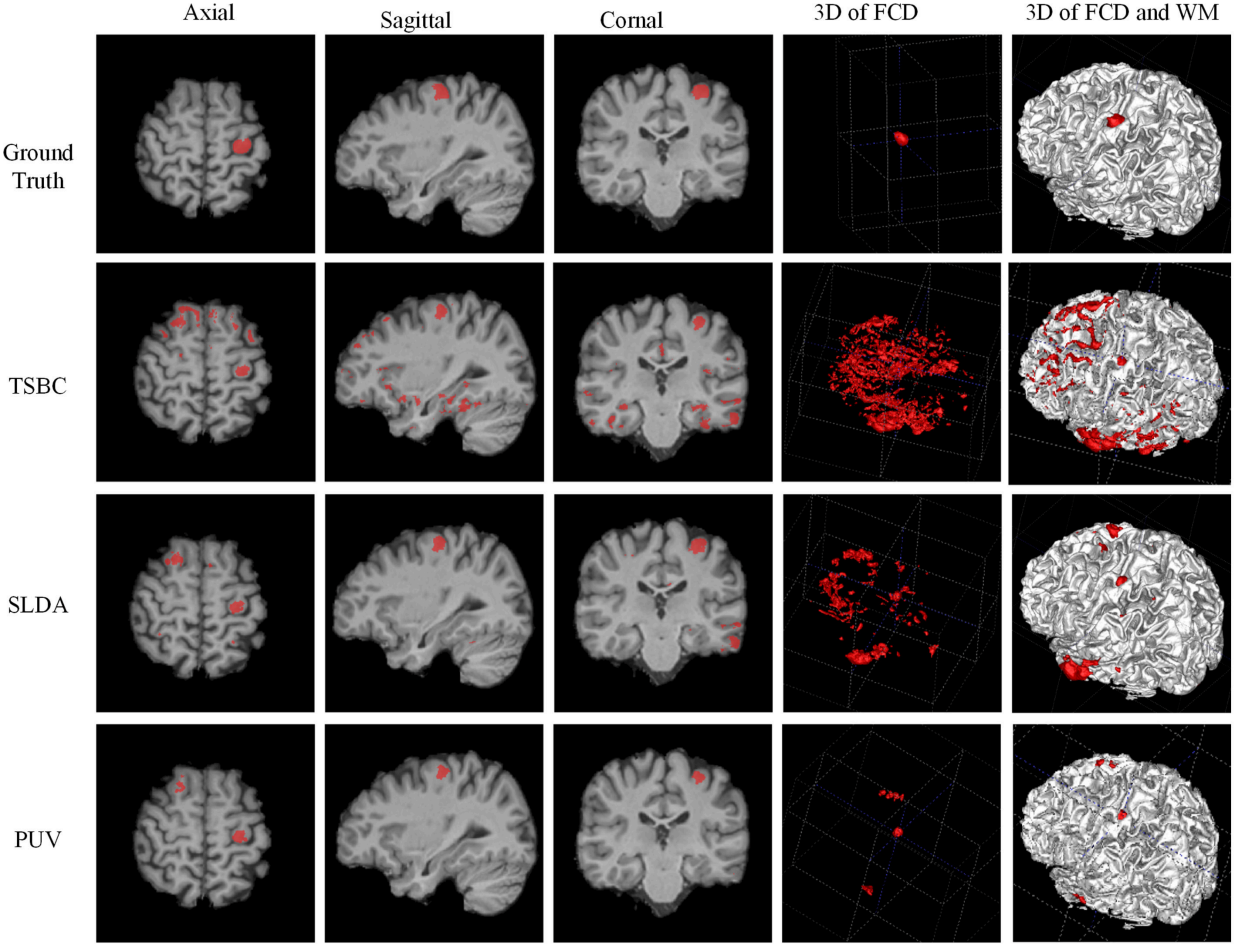
**Examples of classification results of the 3D image from patient 8 with FCD lesions**. From left to right rows: axial, sagittal, coronal, and two 3D views. In the ground truth image (the first row), the FCD regions are in red. From the results of two-stage Bayesian classifiers, surface-based LDA, and PUV methods (from second row to the bottom row), the voxels and vertex identified as positive (FCD) are also in red.

The experiments were designed to validate features and classifications of the proposed framework. The classifications driven by different feature groups were compared using *F*_s_ to assess the effectiveness of the features, and the results are shown in Figure 9. The proposed method was compared with the NB, LDA, MDA, and QDA classifiers in terms of *F*_s_ (Figure 9) and the vivid classified images (Figure 10) to demonstrate that the proposed method could improve the performances of single classifiers. Moreover, the TSBC (Antel et al., 2003) and SLDA (Hong et al., 2014) methods previously developed for FCD detection were also compared with the proposed method using the evaluation metrics (Table 2) and classified images (Figure 11). Classifications were constructed using the leave-one-out-cross-validation because of the limited MR images. Each test image was classified based on the classifier trained on the rest of the images in the study.

**Table 2:** **Evaluation results of positive unanimous voting (PUV), two-stage Bayesian classifier [3], and surface-based linear discriminant analysis using F-score, true positive rate, and false positive rate**.

	**F**-**score**		**TPR**		**FPR**	
	**Mean**	***SD***	**Mean**	***SD***	**Mean**	***SD***
TSBC	0.0708	0.1131	0.3933	0.2754	0.0536	0.0270
SLDA	0.2060	0.1454	0.5374	0.2979	0.0212	0.0241
PUV1	0.1251	0.1324	0.3008	0.1657	0.0160	0.0091
PUV2	0.3039	0.1865	0.2377	0.1801	0.0211	0.0022

*PUV1 and PUV2 represent the results before and after the connected region size analysis step, respectively*.

The parameters were as follows. The total number of neighboring voxels *N* was set to 27 when the average features of healthy controls were calculated. This value indicated that three voxels were set in each dimension of the 3D images. The local window size for morphological opening *L*_*w*_ was set to 3 in the connected region analysis step. The threshold of the region size was *T*_*s*_ = 900, which was equivalent to the physical size of 112.5mm^3^ = 900 × (0.5*mm*)^3^.

### Comparison of PUV and single classifiers

Figure 9 presents *F*_s_ of the classified images generated from different classifications. Among all feature groups, FG_6_ showed the highest mean *F*_s_, where A_E6_ of FG_6_ was equal to 0.0713, whereas A_*E*1−5_ of FG_1−5_ ranged from 0.0406 to 0.6893. Therefore, FG_6_ was FG_best_ in this study, and subsequent classifications were based on FG_best_.

NB and PUV presented better *F*_s_-values than other methods for FG_1_, FG_2_, and FG_3_. This result indicated that NB and PUV were more suitable for FCD detection than LDA, QDA, and MDA using features that were not compared with the healthy controls. The *F*_s_-values of FG_5_ and FG_6_ using NB and PUV were obviously larger than the other classified results. This characteristic indicated that the feature groups that contained feature difference of GWB width map (*F*_d4_) could improve the performance of FCD detection with a suitable classifier, because FG_5_ and FG_6_ included *F*_d4_, contrary to other feature groups.

Thus, PUV using the FG_6_ provided the best *F*_s_, indicating that the PUV with FG_6_ exhibited better trade-off between precision and recall than other assemblies of feature groups and classifiers in this study.

Figure 10 illustrates the experimental results from MDA, QDA, LDA, NB, and PUV driven by FG_best_. All classification methods correctly identified the true FCD as positive on the 2D images (axial, sagittal, and coronal views) compared with the ground truth, but different numbers of FP were also obtained. In addition, PUV could reduce the FP regions as shown by the fourth column in Figure 10, because it had the lowest number of color regions in 3D views among all classifications in this study. This phenomenon indicated that PUV facilitated the identification of the true FCD regions among positive results.

### Comparison of PUV and two existing methods of FCD detection

Figure 11 demonstrates a comparison of PUV, TSBC, and SLDA. All the three methods successfully classified the FCD region as positive from the axial, sagittal, and coronal views. SLDA and TSBC produced significantly more FP results than PUV using the 3D images. This result indicated that PUV could reduce the FP results, such that the TP regions were easy to delineate from all voxels identified as positive by a classifier.

Table 2 describes the evaluation results from the *F*_*s*_, TPR, and FPR-values of PUV1, PUV2, TSBC, and SLDA. PUV1 and PUV2 had lower mean TPRs than TSBC and SLDA, because the combination of multiple classifiers relabeled parts of the true lesional region as negative when reducing the FP results.

PUV2 showed a larger mean *F*_s_ than TSBC and SLDA, whereas PUV1 had a larger mean *F*_s_ than TSBC but lower mean *F*_s_ than SLDA. This phenomenon suggested that the connected region analysis step could help improve the trade-off between precision and recall of FCD detection. The *SD*-values of *F*_s_ were large for all methods relative to the mean values, because the size of the lesions had larger ranges from 499 to 23,667 mm^3^ (μ±σ was 4179 ± 6598) in this study. For example, the lesional size of patient number 10 was 23,667 mm^3^, which was large. The *Fs*-values of this patient were 0.38 for TSBC, 0.45 for SLDA, and 0.69 for PUV2. By contrast, the lesional size of patient number 3 was 1754 mm^3^, which was small. The *Fs*-values of this patient were 0.04 for TSBC, 0.06 for SLDA, and 0.28 for PUV2.

PUV2 provided lower mean FPR and considerably lower *SD*-values of the FPR than TSBC. This result suggested that the proposed framework had lower and more stable probability of falsely classifying non-lesional regions as positives than TSBC. PUV2 provided similar mean FPR but smaller *SD*-values of the FPR compared with SLDA. Thus, PUV2 presented comparable ability of correctly classifying non-lesional regions as SLDA, but the ability of PUV2 was remarkably more stable than that of SLDA.

### Results of connected region analysis and evaluation on subject level

**Figure 12:**
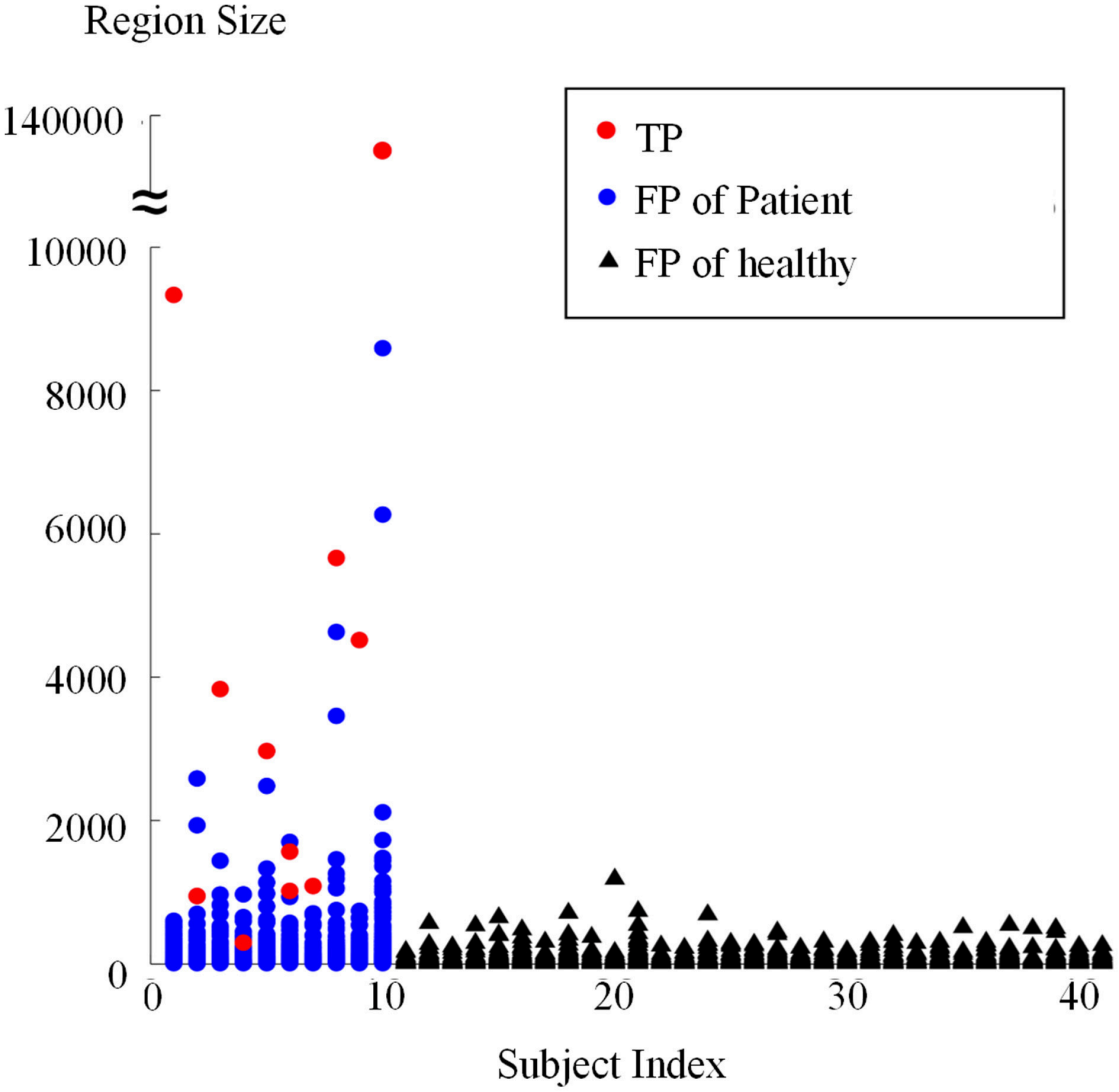
**Sizes of regions classified as positive from patients and healthy controls using connected region analysis**. The red dots are the region size values of the true FCD lesions, blue dots are the region sizes of FP regions in patients, and black triangles are the region sizes of FP regions of healthy controls.

Connected region analysis extended the classification results from the voxel level to the subject level (Figure 12). Each subject, even the healthy controls (black triangles), had voxels falsely classified as lesional. However, most true lesional regions were larger than the falsely classified regions in healthy controls (red dots vs. black triangles), confirming that relabeling the small positive regions as negative was reasonable. After relabeling all small/tiny positive regions smaller than *T*_*s*_ (size of 900 voxels or 112.5 mm^3^) as negative, eight out of 10 patients were correctly identified as patients, whereas 30 of 31 healthy controls were correctly classified as healthy. Thus, TPR was 80% and FPR was 3.3% on the subject level, indicating that the proposed framework produced promising results for FCD detection.

### Limitations

This study was limited by four factors. First, region size analysis possibly split the TP regions. For example, two red dots are shown in Figure 12 when the subject index was 6, which indicated that the true lesional region of patient number 6 was split into two clusters. In future studies, methods of splitting and merging of regions might address this issue. Second, this study used a limited number of single classifiers. Including more effective single classifiers might also further improve automatic FCD detection. Third, the proposed framework was affected by registration accuracy, because the feature difference step in the proposed framework compared images from different subjects and the volumes of images remarkably varied in different subjects. PUV and SLDA both depended on registration accuracy, contrary to TSBC. However, PUV and SLDA produced better performance for FCD detection. Thus, comparing registered images from different subjects was necessary, although the comparative results were dependent on registration. Fourth, the detection performance is affected by the intensity normalization, since the feature computation are related to the intensity values. When the images are obtained from different scanners, the intensity scale should be carefully normalized and the intensity non-uniformity has to be carefully adjusted. Using good evaluation methods to guarantee the intensities are properly corrected, might further improve the detection performance.

## Conclusion

In this study, we proposed a new framework based on PUV to achieve automated detection of FCD lesions. Feature determination of the proposed method enhanced the contrasts between lesional and non-lesional regions. Selection of feature group found FG_best_ in this study. Unanimous voting for feature classification categorized image voxels into positives or negatives. Finally, the connected region refined the classified images through relabeling the small fragment region as negative.

Experiments on the small number of the FCD samples reveal that the proposed framework produced higher *F*_s_-values and lesser number of FP regions compared with single classifier techniques, such as NB, LDA, QDA, and MDA, facilitating the easy identification of true FCD lesions. The proposed framework had lower and more stable probability of falsely classifying non-lesional regions as positives compared with TSBC. The proposed framework presented comparable ability of correctly classifying the non-lesional regions compared with SLDA. However, the ability of the proposed work was much more stable. After tiny fragments were relabeled as negative using connected region analysis, 8/10 patients and 30/31 healthy controls were correctly recognized.

The intensity scale standardization and intensity non-uniformity correction might affect the generalizability of the proposed method when the images are over scanners with variable intensity bias patterns. In the future, to apply the proposed method on more data sets that from different scanners, intensity scale standardization and intensity non-uniformity correction should be cautiously operated before tissue segmentation and feature computation. The reason is that two limitations of the proposed work are non-ignorable: (1) the method is tested on the small number of FCD samples; (2) the images are from same scanner. The intensity scale standardization and intensity non-uniformity correction on the data sets from different canners, might change the intensities of different brain tissue. The tissue segmentation and feature computation highly depend on the intensities of tissues, and directly affect the detection performance. Therefore, the proposed method might not provide detection results as good as this study when more data sets from different canners are considered, due to the preprocessing in terms of intensity scale standardization and intensity non-uniformity correction may change the information of tissues.

Based on the experiments in this study, the proposed work was simple, easy to reproduce, and may become a useful tool to assist doctors in detecting and diagnosing FCD lesions.

## Author contributions

XQ performed the experiments, analyzed the data sets and wrote the manuscript. JY designed the work and finalized this manuscript. SM modified the manuscript and checked all the figures and texts. WP and TB designed the topic of the work and provided the framework of the topic.

### Conflict of interest statement

The authors declare that the research was conducted in the absence of any commercial or financial relationships that could be construed as a potential conflict of interest.

## References

[B1] Antel S. B. Bernasconi A. Bernasconi N. Collins D. L. Kearney R. E. Shinghal R.. (2002). Computational models of MRI characteristics of focal cortical dysplasia improve lesion detection. Neuroimage 17, 1755–1760. 10.1006/nimg.2002.1312 12498749

[B2] Antel S. B. Collins D. L. Bernasconi N. Andermann F. Shinghal R. Kearney R. E.. (2003). Automated detection of focal cortical dysplasia lesions using computational models of their MRI characteristics and texture analysis. Neuroimage 19, 1748–1759. 10.1016/S1053-8119(03)00226-X 12948729

[B3] Bernasconi A. Antel S. B. Collins D. L. Bernasconi N. Olivier A. Dubeau F.. (2001). Texture analysis and morphological processing of magnetic resonance imaging assist detection of focal cortical dysplasia in extra-temporal partial epilepsy. Ann. Neurol. 49, 770–775. 10.1002/ana.1013 11409429

[B4] Bernasconi A. Bernasconi N. (2011). Advances in MRI for ‘cryptogenic’ epilepsies. Nat. Rev. Neurol. 7, 99–108. 10.1038/nrneurol.2010.199 21243016

[B5] Besson P. Bernasconi N. Colliot O. Evans A. Bernasconi A. (2008a). Surface-based texture and morphological analysis detects subtle cortical dysplasia. Med. Image Comput. Comput. Assist. Interv. 11, 645–652. 10.1007/978-3-540-85988-8_77 18979801

[B6] Besson P. Colliot O. Evans A. Bernasconi A. (2008b). Automatic detection of subtle focal cortical dysplasia using surface-based features on MRI, in The 5th IEEE International Symposium on Biomedical Imaging (ISBI): From Nano to Macro (Paris).

[B7] Colliot O. Antel S. B. Naessens V. B. Bernasconi N. Bernasconi A. (2006). *In vivo* profiling of focal cortical dysplasia on high-resolution MRI with computational models. Epilepsia 47, 134–142. 10.1111/j.1528-1167.2006.00379.x 16417541

[B8] Despotovic I. Segers I. Platisa L. Vansteenkiste E. Pizurica A. Deblaere K.. (2011). Automatic 3D graph cuts for brain cortex segmentation in patients with focal cortical dysplasia. Conf. Proc. IEEE Eng. Med. Biol. Soc. 2011, 7981–7984. 10.1109/IEMBS.2011.6091968 22256192

[B9] Diedrichsen J. Balsters J. H. Flavell J. Cussans E. Ramnani N. (2009). A probabilistic MR atlas of the human cerebellum. Neuroimage 46, 39–46. 10.1016/j.neuroimage.2009.01.045 19457380

[B10] Duda R. O. Hart P. E. Stork D. G. (2000). Pattern Classification, 2nd Edn. New York, NY: John Wiley & Sons, Inc.

[B11] Hong S. J. Kim H. Schrader D. Bernasconi N. Bernhardt B. C. Bernasconi A. (2014). Automated detection of cortical dysplasia type II in MRI-negative epilepsy. Neurology 83, 48–55. 10.1212/WNL.0000000000000543 24898923 PMC4114179

[B12] Huppertz H. Grimm C. Fauser S. Kassubek J. Mader I. Hochmuth A.. (2005). Enhanced visualization of bluffed gray-white matter junctions in focal cortical dysplasia by voxel-based 3D MRI analysis. Epilepsy Res. 67, 35–50. 10.1016/j.eplepsyres.2005.07.009 16171974

[B13] Kassubek J. Huppertz H. Spreer J. Schulze-Bonhage A. (2002). Detection and localization of focal cortical dysplasia by voxel-based 3D MRI analysis. Epilepsia 43, 596–602. 10.1046/j.1528-1157.2002.41401.x 12060018

[B14] Loyek C. Woermann F. G. Nattkemper T. W. (2008). Detection of Focal Cortical Dysplasia Lesions in MRI Using Textural Features. Berlin; Heidelberg: Springer.

[B15] Mazziotta J. Toga A. Evans A. Fox P. Lancaster J. Zilles K.. (2001). A probabilistic atlas and reference system for the human brain: International Consortium for Brain Mapping (ICBM). Philos. Trans. R. Soc. Lond. B Biol. Sci. 356, 1293–1322. 10.1098/rstb.2001.0915 11545704 PMC1088516

[B16] Nyul L. G. Udupa J. K. (1999). On standardizing the MR image intensity scale. Magn. Reson. Med. 42, 1072–1081. 10571928 10.1002/(sici)1522-2594(199912)42:6<1072::aid-mrm11>3.0.co;2-m

[B17] Qu X. Platisa L. Despotovic I. Kumcu A. Bai T. Deblaere K.. (2014). Estimating blur at the brain gray-white matter boundary for FCD detection in MRI. Conf. Proc. IEEE Eng. Med. Biol. Soc. 2014, 3321–3324. 10.1109/EMBC.2014.6944333 25570701

[B18] Rajan J. Kannan K. Kesavadas C. Thomas B. (2009). Focal Cortical Dysplasia (FCD) lesion analysis with complex diffusion approach. Comput. Med. Imaging Graph. 33, 553–558. 10.1016/j.compmedimag.2009.05.004 19560319

[B19] Smith S. M. (2002). Fast robust automated brain extraction. Hum. Brain Mapp. 17, 143–155. 10.1002/hbm.10062 12391568 PMC6871816

[B20] Wozniak M. Grana M. Corchado E. (2014). A survey of multiple classifier systems as hybrid systems. Inform. Fusion 16, 3–17. 10.1016/j.inffus.2013.04.006

[B21] Zhang Y. Brady M. Smith S. (2001). Segmentation of brain MR images through a hidden Markov random field model and the expectation-maximization algorithm. IEEE Trans. Med. Imaging 20, 45–57. 10.1109/42.906424 11293691

